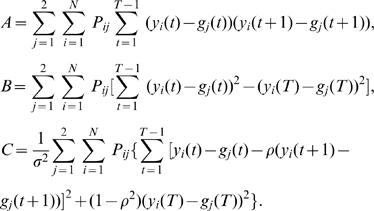# A Computational Approach for Functional Mapping of Quantitative Trait Loci That Regulate Thermal Performance Curves

**DOI:** 10.1371/journal.pone.0000554

**Published:** 2007-06-20

**Authors:** John Stephen Yap, Chenguang Wang, Rongling Wu

**Affiliations:** Department of Statistics, University of Florida, Gainesville, Florida, United States of America; University of Chicago, United States of America

## Abstract

Whether and how thermal reaction norm is under genetic control is fundamental to understand the mechanistic basis of adaptation to novel thermal environments. However, the genetic study of thermal reaction norm is difficult because it is often expressed as a continuous function or curve. Here we derive a statistical model for dissecting thermal performance curves into individual quantitative trait loci (QTL) with the aid of a genetic linkage map. The model is constructed within the maximum likelihood context and implemented with the EM algorithm. It integrates the biological principle of responses to temperature into a framework for genetic mapping through rigorous mathematical functions established to describe the pattern and shape of thermal reaction norms. The biological advantages of the model lie in the decomposition of the genetic causes for thermal reaction norm into its biologically interpretable modes, such as hotter-colder, faster-slower and generalist-specialist, as well as the formulation of a series of hypotheses at the interface between genetic actions/interactions and temperature-dependent sensitivity. The model is also meritorious in statistics because the precision of parameter estimation and power of QTLdetection can be increased by modeling the mean-covariance structure with a small set of parameters. The results from simulation studies suggest that the model displays favorable statistical properties and can be robust in practical genetic applications. The model provides a conceptual platform for testing many ecologically relevant hypotheses regarding organismic adaptation within the *Eco-Devo* paradigm.

## Introduction

Understanding the genetic variation of phenotypic responses to a range of environments (referred to as phenotypic plasticity or reaction norm) and its impact on selection and evolution has been a central challenge for studies in evolutionary genetics and ecology [1]–[3]. Environment-dependent responsiveness of a genotype can be broadly classified into two types in terms of whether the environment is discrete or continuous. While the mechanistic basis for the phenotypic plasticity of a genotype to discrete environments has been extensively investigated [1]–[6], we know almost nothing about the pattern of phenotypic expression of a single genotype across continuous environmental gradients, such as temperature and humidity gradients [5]. Virtually, many biological traits vary continuously when the environmental state is continuous, for which the phenotypic value of a trait can be expressed as a function of the environmental states. These traits are often called “infinite-dimensional” traits that require an infinite number of measurements to be completely described [6]. Thermal performance curves (TPCs) which are of evolutionary significance present one of such excellent examples [7]–[10]. TPCs represent the change in performance of an individual or a genotype as a function of temperature.

**Figure 1:**
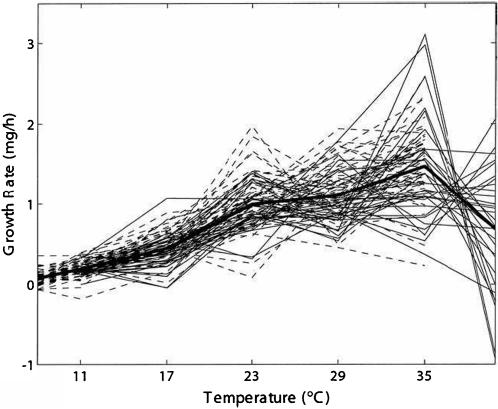
Example of thermal performance curves – growth rate z was measured at six temperatures for 90 families of caterpillars. This set of curves has a common shape (slowly increases, tend to reach a maximum and rapidly decreases). The variation in the curves is due to both genetic and environmental factors operational in the population. Adapted from ref. [10].

Figure 1 illustrates the growth rate of caterpillars, *y*, measured at six temperatures ranging from 11° Celsius to 40° Celsius, that is, (11, 17, 23, 29, 35, 40) [10]. Although the data are discontinuous, the underlying relationship between the growth rate and the temperature (*t*) can be described by a mathematical function, such as high-order polynomials, that is biologically meaningful or statistically justifiable [10]. In general, the TPC curve slowly increases with increasing temperature, tends to reach a maximum at some intermediate temperature and then rapidly decreases with further increase in temperature, but it is obvious that there exists pronounced differentiation in curve shape among individuals due to genetic and environmental effects. One purpose of the development of statistical models is to separate these two different types of effects on TPCs and test their relative importance in governing the shape of the curves. The second purpose is to address an important question about the genetic architecture of continuous reaction norms: specifically, what are the patterns of genetic variation and covariation in continuous reaction norms found in natural populations [2]?

**Figure 2:**
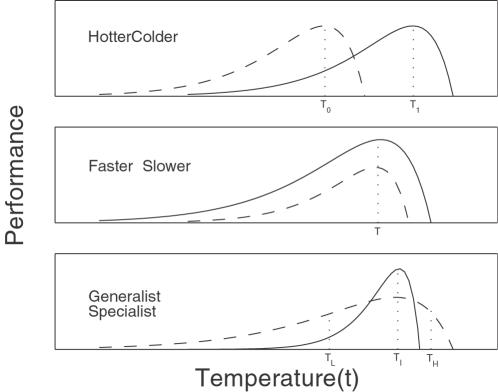
Three hypothetical patterns of variation in thermal performance curves due to the effects of a hotter/colder, faster/slower and genegeneralist/specialist QTL, respectively.

Three distinct modes of variation have been proposed to describe the variation of TPCs, i.e., hotter-colder, faster-slower and generalist-specialist [8] (Fig. 2). Specifically, these modes are defined as follows:

1. The *hotter-colder* describes variation in the temperature at which performance is maximal, in which some individuals (hotter, 1) have maximal performance at hotter temperatures (τ_1_), whereas others (colder, 0) have maximal performance at colder temperatures (τ_0_) relative to the mean reaction norm for the population.
2. The *faster-slower* captures variation in the overall height of the reaction norm, in which some individuals (faster, 1) have greater performance at all temperatures than others (slower, 0).
3. The *generalist-specialist* shows variation in the width of the reaction norm, in which individuals with greater performance at intermediate temperatures (τ*_I_*) have lower performance at low (τ*_L_*) and high temperatures (τ*_H_*) (specialists, 1), whereas individuals with lower performance at intermediate temperatures have greater performance at low and high temperatures (generalists, 0) relative to the mean reaction norm for the population.

Each of these three patterns, hotter-colder, faster-slower, and generalist-specialist, that can be viewed as different directions of variation may be controlled by a particular set of genes. It is possible that these three sets of genes may exist simultaneously in the same population such that this population may contain mixtures of genotypes that vary along different axes of variation [11]. A central challenge is to test how specific genes govern each mode of variation and quantify how much different modes of variation contribute to the total genetic variation for TPCs in a population [12].

The questions mentioned above can now be addressed by using genetic mapping approaches [13]–[16] that capitalizes on molecular markers to infer the underlying quantitative trait loci (QTL) for thermal reaction norms. However, these traditional approaches can only be well used to associate marker genotypes with single phenotypic values of a trait, and have less power to map a phenotype, such as thermal reaction norms, expressed as an “infinite-dimensional” curve. Although extensions have been made to model multiple discrete traits at the same time [17], [18], they are limited for mapping a large number of traits due to computational prohibition and longitudinal repeated measures showing an autocorrelation structure. More recently, a new QTL mapping strategy, called functional mapping, has been proposed to map traits that vary continuously as a function of an independent variable [19], [20]. By embedding biologically sensible growth equations into the mapping framework, functional mapping can estimate the dynamic changes of the genetic effects of a QTL in development and push hypotheses tests towards the interplay between genes and development.

In principle, functional mapping can be used to study the genetic architecture of environmentally sensitive phenotypic variation for a complex trait. However, a direct use of functional mapping is problematic because it has not taken into account the underlying modes of variation unique to thermal reaction norms [8]. The purpose of this study is to derive a theoretical framework model for mapping QTL that regulate differentiation in TPC described by a rational function. By testing the mathematical parameters that define the optimum performance breadth and thermal limit of a TPC, a general procedure is given for testing and identifying possible existence of a particular underlying mode of variation. The model allows for a further extension to discern the contributions of multiple modes of variation to TPCs through a web of genetic actions and interactions. The model and procedure are derived within the maximum likelihood context and implemented with the EM algorithm. Monte Carlo simulation studies are performed to explore the statistical properties of the model and validate its usefulness in practice.

## Method

### Mixture Model

To simplify the description of the model, we assume a backcross population in which there are only two contrast genotypes at each locus. The model can be readily extended to other more complicated designs, such as the F_2_, a full-sib family, a natural population and a structured pedigree with multiple founders. The backcross considered has *n* individuals, each genotyped with polymorphic markers for the construction of a linkage map. This map is used to identify the genome-wide distribution of QTL that control TPCs. All the backcross individuals are subjected to a multitude of temperature (say *T*), which cover the range suited for the species studied to grow normally. At each temperature, body mass or body size (in terms of length, width or volume) of the backcross is measured at multiple time points, from which the mean rate of growth is calculated. Thus, the relationship between growth rate and temperature describe the TPC which is modeled by a rational function.

Suppose there is a putative QTL segregating with two different genotypes *Qq* (coded by 1) and *qq* (coded by 0) in the assumed backcross that affects the shape of TPCs. This QTL is located somewhere in the genome, which can be detected by the linkage map. Assume the QTL to reside between a pair of flanking markers **M**_1_ and **M**_2_ each with two genotypes coded by 1 and 0. For each backcross individual, it may carry one (and only one) QTL genotype, 1 or 0. The probability of a particular individual (*i*) to carry QTL genotype 1 or 0 depends on the marker genotype of this individual at two flanking markers (**M**_1_ and **M**_2_) that bracket the QTL. Let *r*_1_, *r*_2_ and *r* be the recombination fractions between **M**_1_ and QTL, between QTL and **M**_2_ and between the two markers, respectively. Under the assumption of independent crossovers, we derive the probability of a QTL genotype given a marker genotype as
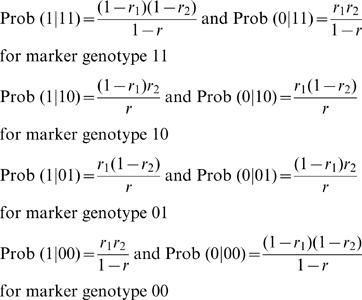
Because each individual has a known marker genotype, 11, 10, 01 or 00, these conditional probabilities are generally expressed by ω_1|*i*_ and ω_0|*i*_.

The phenotypic value of growth rate for individual *i* at different temperatures, **y** *_i_* = (*y_i_*(1),…,*y_i_*(*T*)), is distributed as a mixture distribution with two different groups of QTL genotypes, expressed as
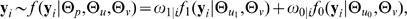
(1)where Θ*_p_* = (ω_1|*i*_, ω_0|*j*_) is the vector of individual-specific mixture proportions (i.e., the conditional probabilities of QTL genotypes) which are constrained to be non-negative and sum to unity, 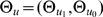 where 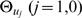 is a vector that contains the parameters specific to component (i.e., QTL genotype) *j*, and Θ*_v_* includes the parameters common to all components. We assume that given the *i*th individual's QTL genotype *j*, its repeated measures follow a multivariate normal distribution, expressed as
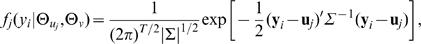
where **u** *_j_* = (*u_j_*(1),…,*u_j_*(*T*)) is a vector of expected values for QTL genotype *j* at different temperatures. At a particular temperature *t*, the relationship between the observation and expected mean can be described by a regression model,
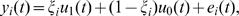
(2)where ξ*_i_* is the indicator variable denoted as 1 for *j* = 1 and 0 for *j* = 0, and *e_i_*(*t*) is the residual error (i.e., the accumulative effect of polygenes and errors) that is independently and identically distributed (iid) normal with mean zero and variance σ^2^(*t*). The errors at two different time points or states, *t*_1_ and *t*_2_, are correlated with covariance σ(*t*_1_, *t*_2_). The covariance matrix Σ is composed of σ^2^(*t*) and σ(*t*_1_, *t*_2_).

### Modeling the Mean-Covariance Structures

Functional mapping models the mean vector and the structure of covariance matrix for longitudinal traits. The genotypic means for growth rate over a range of continuous temperatures can be specified by a biologically meaningful mathematical equation. In a thermal experiment for brown trout, Ojanguren et al. [21] used a third-order polynomial function to sufficiently describe the thermal sensitivity of fish growth. Here, we use a rational function such that a general form of TPC across different temperatures for QTL genotype *j* is expressed as
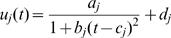
(3)where a combination of *a_j_* and *b_j_* describes the height and base width of the TPC and *c_j_* and *d_j_* describe the horizontal and vertical translation of the curve, respectively. If there are differences in a set of curve parameters, arrayed in 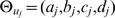, between different genotypes at a QTL, this means that this QTL triggers an effect on TPCs. Further, by estimating different sets of parameters, three modes of variation for TPC, controlled by a hotter-colder, faster-slower and generalist-specialist QTL, respectively, can be elucidated (Fig. 1).

In statistics, theories and methods have been available to model the structure of covariances between measurements repeatedly made at a series of time points [22]. Because of its elegant mathematical and statistical properties, the autoregressive process has been widely used for studies of longitudinal data measurements. The first-order autoregressive (AR(1)) model has been successfully applied to model the structure of the within-subject covariance matrix for functional mapping. The AR(1) model is basedon two simplified assumptions, i.e., variance stationarity – the residual variance (σ^2^) is unchanged over time points, and covariance stationarity – the correlation between different measurements decreases proportionally (in ρ) with increased time interval. Mathematically, the AR(1) is described as
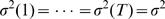
for the variance, and
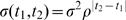
for the covariance between any two time points *t*_1_ and *t*_2_, where 0<ρ<1 is the proportion parameter with which the correlation decays with time lag. The parameters that model the structure of the covariance matrix are arrayed in Θ*_v_* = (ρ, σ^2^).

When the residual covariance matrix (*Σ*) is modeled by the AR(1) model, the closed forms can be derived for its inverse and determinant, which facilitate model computing and parameter estimating. The inverse *Σ* ^−1^ is a tridiagonal symmetric matrix, whose diagonal elements are
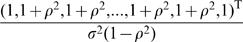
and second diagonal elements are all
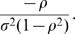
The determinant of the matrix is derived as
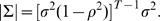
Let **z** *_j_*_|*i*_ = [*z_j_*_|*i*_(1),…,*z_j_*_|*i*_(*T*)] = **y** *_i_*−**u** *_j_*, (*j* = 0,…,*j*), then we have
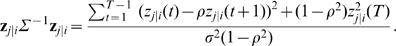
In practice, the two simplified assumptions of the AR(1) model may not hold so that the elegant expressions of the matrix cannot be used for functional mapping. To make longitudinal data well suited to the AR(1) model, some treatments are needed. For example, to remove the heteroscedastic problem of the residual variance, Carroll and Rupert's [23] transform-both-sides (TBS) model is embedded into the growth-incorporated finite mixture model [24], which does not need any more parameters. Both empirical analyses with real examples and computer simulations suggest that the TBS-based model can increase the precision of parameter estimation and computational efficiency. Furthermore, the TBS model preserves original biological means of the curve parameters although statistical analyses are based on transformed data.

The TBS-based model displays the potential to relax the assumption of variance stationarity, but the covariance stationarity issue remains unsolved. Zimmerman and Núñez-Antón [25] proposed a so-called structured antedependence (SAD) model to model the age-specific change of correlation in the analysis of longitudinal traits. The SAD model has been employed in several studies and displays many favorable properties for genetic mapping of dynamic traits [26].

### Likelihood and Estimation

We implemented the EM algorithm, originally proposed by Dempster et al. [27], to obtain the maximum likelihood estimates (MLEs) of three groups of unknown parameters in a QTL mapping model, that is, the conditional probabilities of QTL genotypes (Θ*_p_*) that specify the co-segregation patterns of QTL and markers in a mapping population, the curve parameters 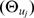 that model the mean vector, and the parameters (Θ*_v_*) that model the structure of the covariance matrix. All these unknowns are contained within the mixture model described by equation (1).

The likelihood of phenotypic values measured at multiple temperatures can be written, in terms of a multivariate mixture model (1), as
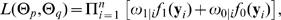
where 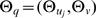 and 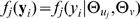. The MLEs of the unknown parameters for a QTL can be computed by implementing the EM algorithm. The log-likelihood is given by
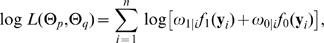
(4)with derivative with respect to any element Θ_ς_ in the unknown vector (*Θ* *_p_*,*Θ* *_q_*)
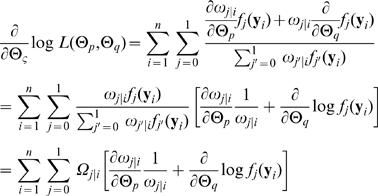
where we define
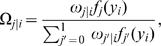
(5)which could be thought of as a posterior probability that individual *i* have QTL genotype *j*. Conditional on 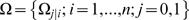, we solve for
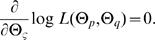
(6)The log-likelihood equations are derived to estimate the parameters in (Θ*_p_*, Θ*_q_*) through the EM algorithm. In the E step, the posterior probabilities of a QTL given marker genotypes and phenotypes observations are calculated with equation (2). Then, in the M step, different parameters are estimated with equation (3). The log-likelihood equations in the M step are given in the Appendix. The iterations between the E and M steps are repeated until the estimates converge. The values at the convergence are regarded as the MLEs. In practice, the QTL position parameter (θ) can be viewed as a fixed parameter because a putative QTL can be searched at every 1 or 2 cM on a map interval bracketed by two markers throughout the entire linkage map. The log-likelihood ratio test statistic for a QTL at a particular map position is displayed graphically to generate a likelihood map or profile. The genomic position that corresponds to a peak of the profile is the MLEof the QTL location.

### Hypothesis Tests

#### Existence of a QTL

The merit of functional mapping includes the tests of a number of biologically meaningful hypotheses regarding the genetic and developmental control of dynamic traits. After the genetic parameters are obtained, we need to test whether there is a QTL that affects the shape of TPC. The existence of a QTL can be tested by formulating the following hypotheses:
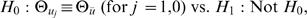
(7)where the null hypothesis *H*_0_ states that the data can be fit with only one mean curve by parameters Θ*_u̅_* = (*a*, *b*, *c*, *d*), whereas in the alternative hypothesis *H*_1_ two distinct curves exist showing that there is a segregating QTL forTPC. The test statistic is the log-likelihood ratio (LR) of the full (*H*_1_) over reduced model (*H*_0_), expressed as
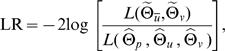
where the tildes and hats denote the MLEs of the unknown parameters under the *H*_0_ and *H*_1_, respectively. Note that the estimation of (*Θ̂* *_p_*, *Θ̂* *_u_*, *Θ̂* *_v_*) depends on both phenotypic values and marker data, whereas the estimation of 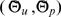 only depends on phenotypic values. The critical threshold for the declaration of a QTL can be determined from permutation tests (Churchill and Doerge 1994).

#### Type of QTL

After a significant QTL for TPCs is identified, the next step is to test how this QTL affects the patterns of TPCs. Three different modes of variation are specified for thermal performance curves [8]. Each of these modes may be controlled by a different gene. The proposed model can be used to identify mode-specific QTL by formulating relevant hypotheses. Whether there is a QTL that controls the hotter-colder variation can be tested on the basis of the following hypotheses
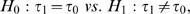
where τ_1_ and τ_0_ are the temperatures at which the TPC reaches a maximum value for QTL genotype 1 and 0, respectively. These temperatures corresponding to the maximum performance can be obtained by solving the following equations
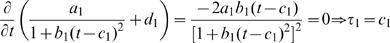
and
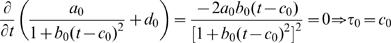
Thus, testing hypothesis (8) is equivalent to testing the hypothesis
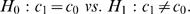
The QTL for the faster-slower mode of variation can be detected by
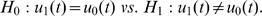
(8)Although the alternative hypothesis of (1) contains two possibilities *u*_1_(*t*)>*u*_0_(*t*) or *u*_1_(*t*)<*u*_0_(*t*), the property of a rational function indicates that only one possibility exists consistently at all temperatures during the entire range. Thus, the rejection of the null hypothesis suggests that one QTL genotype performs better at all temperatures than the second genotype.

The identification of a so-called generalists-specialist QTL is more difficult, compared with that of the hotter-colder and faster-slower QTL. First, by solving the equation *u*_1_(*t*) = *u*_0_(*t*), we obtain the two temperatures, denoted by 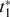 and 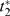 (assuming 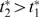), at which the two QTL genotypic TPCs cross over. Second, based on these two temperatures, the TPC is divided into three distinct regions of temperature 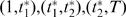. The hypotheses for detecting the generalist-specialist QTL are made on the basis of the area under curve, i.e.,
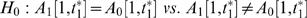
(9)

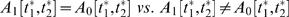
(10)

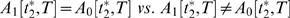
(11)where
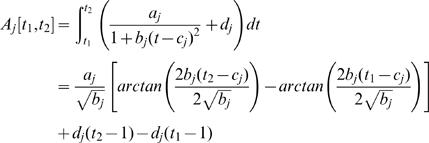
The rejection of each null hypothesis from (9) to (11) indicates the existence of a generalists-specialist QTL.

Different from the hypothesis test about the existence of a QTL (7), there is no problem of non-identifiability for hypothesis tests (8)–(11). Thus, the log-likelihood test statistics calculated for the hypotheses tests (8)–(11) can be reasonably assumed to asymptotically follow a χ^2^-distribution with the degree of freedom equal to the difference in the number of parameters to be estimated under the null and alternative hypotheses.

### Monte Carlo Simulation

#### Design

We performed simulation studies to investigate the statistical behavior of the proposed model. A backcross design with two genotypes 1 and 0 at each locus is simulated. We simulated 10 equally spaced markers, with the recombination fraction of *r* = 0.2 apart, to construct a linkage map of 229.87 cM. Assume that a putative QTL is located at 5.27 cM from the second marker (with the recombination fraction (*r*_1_ = 0.05) between the second marker and QTL). The first marker was randomly generated using Bernoulli (0.5). The succeeding markers were randomly generated using Bernoulli (*p*) where *p* depends on the genotype of the previous marker; that is, if the previous marker genotype was 0, then the next marker genotype was Bernoulli (*r*) and ifit was 1, then Bernoulli (1−*r*). The QTL was generated using Bernoulli (*r*_1_) if the second marker genotype was 0 and Bernoulli (1−*r*_1_), if it was genotype 1.

The phenotypic values for TPCs are simulated by summing the QTL genotypic curves and multivariate-normally distributed residual errors with mean vector zero and covariance matrix Σ structured by the AR(1) model. The genotypic TPCs are assumed separately for different modes of variation in temperature-dependent performance, hotter-colder, faster-slower and generalist-specialist. Each mode corresponds to the control of a different QTL accordingly defined as the hotter-colder, faster-slower and generalist-specialist QTL. The TPC parameters that specify each of these modes for different types of QTL were chosen from the space of these curve parameters (see an example in Fig 1). The simulation studies were designed for different sample sizes (*n* = 100 and 400) and different heritabilities (*H* ^2^ = 0.1 and 0.4). The covariance-structuring AR(1) parameters are given to assure the heritability of the phenotypic values at the middle temperature at *H* ^2^ = 0.1 and 0.4.

#### Results

**Figure 3:**
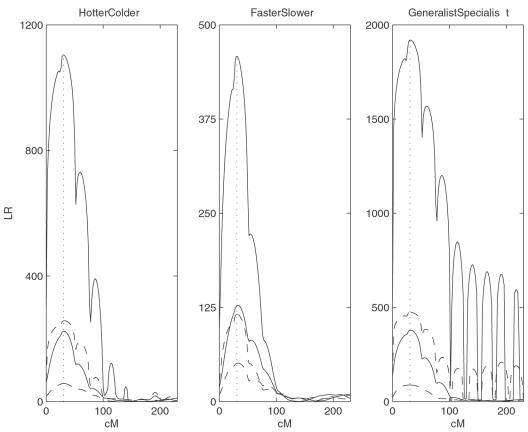
LR plotted over the interval of markers. Solid curves correspond to *n* = 400 whereas broken curves to *n* = 100. Higher curves for each *n* correspond to higher heritability. The vertical dotted line shows the true location of the QTL at 30.81 cM from the first marker.

The simulated marker and phenotypic TPC data are analyzed by the proposed model. By assuming a putative QTL at every 2 cM on the simulated linkage group, the log-likelihood ratio test statistics (LR) calculated for hypotheses (7) were plotted (Fig. 3). The peak of the LR profile corresponds to the MLE of the QTL location. The critical value for declaring the existence of a QTL was determined from 100 permutation tests.

The means and standard errors (SEs) of the MLEs of the QTL location, genotype-specific curve parameters, and covariance-structuring parameters were calculated from 100 repeated simulations (Table 1). In general, the proposed model can provide a reasonable estimate of the QTL location for different modes of variation. But there is the best estimation precision for the location of the generalist-specialist QTL, followed by the hotter-colder and faster-slower QTL, although such difference disappears for a large sample size and heritability (Table 1). As expected, the estimation accuracy and precision of the QTL location increase exponentially with increasing sample sizes and heritability levels of TPC for all the modes of variation.

**Table 1:** The averaged MLEs of the QTL position, curve and AR(1) parameters and their standard errors (given in parentheses) for different QTL types under different sample sizes (*n*) and heritabilities (*H* ^2^) based on 100 simulation replicates

Mode	*H* ^2^	*n*	QTL Location	QTL genotype 1				QTL genotype 0				AR(1) parameter	
				*â*_1_	*bˆ*_1_	*ĉ*_1_	*dˆ*_1_	*â*_0_	*bˆ*_0_	*ĉ*_0_	*dˆ*_0_	σˆ^2^	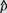
Hotter-colder	0.1	100	29.74 (6.97)	1.02 (0.10)	0.21 (0.07)	5.00 (0.15)	10.00 (0.10)	1.03 (0.12)	0.21 (0.06)	3.49 (0.15)	9.98 (0.11)	0.22 (0.01)	0.60 (0.03)
	0.1	400	30.68 (2.21)	1.01 (0.05)	0.20 (0.03)	5.01 (0.06)	10.00 (0.05)	1.01 (0.06)	0.20 (0.03)	3.50 (0.06)	10.00 (0.05)	0.22 (0.01)	0.60 (0.01)
	0.4	100	31.00 (2.88)	1.01 (0.04)	0.20 (0.02)	5.00 (0.05)	10.00 (0.03)	1.01 (0.04)	0.20 (0.02)	3.50 (0.06)	9.99 (0.04)	0.04 (0.00)	0.59 (0.03)
	0.4	400	30.86 (1.37)	1.00 (0.02)	0.20 (0.01)	5.00 (0.02)	10.00 (0.02)	1.00 (0.02)	0.20 (0.01)	3.50 (0.02)	10.00 (0.02)	0.04 (0.00)	0.60 (0.01)
Faster- slower	0.1	100	31.10 (6.90)	1.15 (0.83)	0.22 (0.12)	4.99 (0.26)	10.37 (0.86)	1.06 (0.19)	0.23 (0.11)	4.95 (0.23)	9.96 (0.18)	0.56 (0.04)	0.60 (0.03)
	0.1	400	30.34 (3.16)	1.02 (0.09)	0.20 (0.05)	5.01 (0.10)	10.49 (0.08)	1.01 (0.09)	0.21 (0.06)	4.97 (0.12)	10.00 (0.08)	0.56 (0.02)	0.60 (0.01)
	0.4	100	30.74 (3.10)	1.01 (0.07)	0.20 (0.03)	5.00 (0.09)	10.50 (0.05)	1.01 (0.07)	0.21 (0.04)	4.97 (0.09)	10.00 (0.06)	0.09 (0.01)	0.59 (0.03)
	0.4	400	30.74 (1.38)	1.00 (0.03)	0.20 (0.02)	4.99 (0.04)	10.50 (0.03)	1.00 (0.03)	0.20 (0.02)	5.00 (0.04)	10.00 (0.03)	0.09 (0.00)	0.60 (0.01)
Generalist- specialist	0.1	100	30.22 (4.38)	1.25 (0.06)	0.61 (0.08)	5.00 (0.06)	10.00 (0.04)	1.02 (0.14)	0.11 (0.04)	4.96 (0.16)	9.98 (0.14)	0.14 (0.01)	0.60 (0.03)
	0.1	400	30.64 (2.07)	1.25 (0.03)	0.60 (0.04)	5.00 (0.03)	10.00 (0.02)	1.00 (0.08)	0.10 (0.02)	4.98 (0.09)	10.00 (0.08)	0.14 (0.00)	0.60 (0.01)
	0.4	100	30.72 (2.63)	1.25 (0.03)	0.60 (0.03)	5.00 (0.02)	10.00 (0.02)	1.01 (0.05)	0.10 (0.01)	4.99 (0.06)	9.99 (0.05)	0.02 (0.00)	0.59 (0.02)
	0.4	400	30.88 (1.31)	1.25 (0.01)	0.60 (0.01)	5.00 (0.01)	10.00 (0.01)	1.00 (0.03)	0.10 (0.01)	4.99 (0.03)	10.00 (0.03)	0.02 (0.00)	0.60 (0.01)

The residual covariance matrix is modeled by correlation ρ = 0.6 and variance σ^2^ = 0.22/0.04 for the hotter-colder QTL, 0.56/0.09 for the faster-slower QTL and 0.14/0.02 for the generalist-specialist QTL when the heritability of TPCs is 0.1/0.4, respectively.

**Figure 4:**
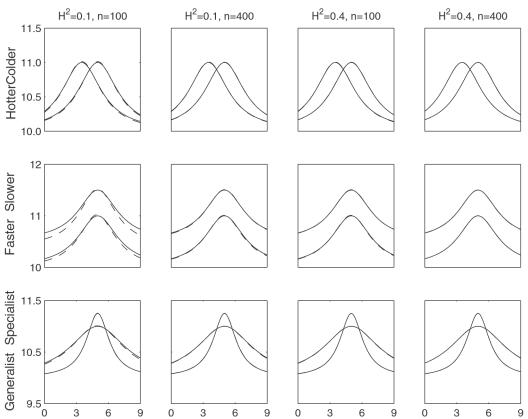
Estimated (solid) and given (broken) TPCs for two different QTL genotypes at different types of QTL under different samples and heritabilities. The given curves for two different QTL genotypes are specified by 
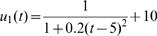
 and 
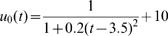
 for the hotter-colder gene; 
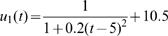
 and 
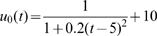
 for the faster-slower gene and 
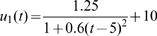
 and 
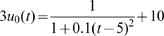
 for the generalist-specialist gene. In many cases, the estimated curves overlap with the given curves, suggesting that the model provides an unbiased estimate of TPCs. Curve parameters defined to specify three different modes of variation in TPC each controlled by a different QTL type in a backcross population.

Although the model can reasonably estimate the curve and AR(1) parameters, the accuracy and precision of estimation depend heavily upon the mode of variation (Table 1). Figure 4 illustrates the comparisons between the estimated and given TPCs for different QTL genotypes from each mode of variation. The estimation of the faster-slower mode is least precise, whereas the generalist-specialist mode has the best estimation precision, with the hotter-colder mode intermediate. For all the modes,a modest sample size (100) and heritability (0.1) can be sufficient to estimate the parameters of TPC curves, but increasing sample sizes and heritabilities are always favorable to improve the precision of parameter estimation.

**Figure 5:**
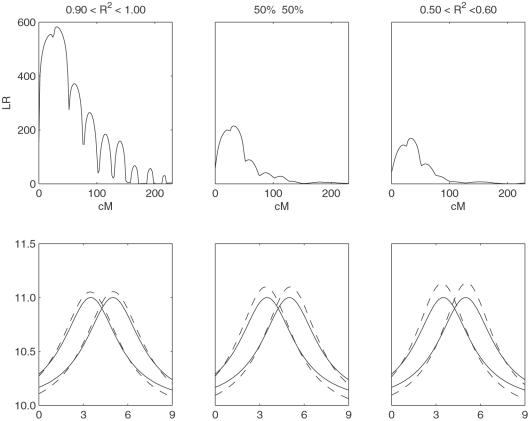
Results from simulation scenarios for a hotter-colder QTL. Scenario 1–the coefficients of determination (*R* ^2^) equal to 0.9–1.0 for all individuals; Scenario 2–*R* ^2^ = 0.9–1.0 for a half of individuals and *R* ^2^ = 0.5–0.6 for the other half; Scenario 3–*R* ^2^ = 0.5–0.6 for all individuals. Upper panel: LR plotted over the interval of markers. Lower panel: Estimated (solid) and given (broken) TPCs for two different QTL genotypes.

An additional simulation study was conducted to examine how poorly TPC-fitted data affect the estimates of model parameters. We simulated TPC data for the hotter-colder gene by considering three different scenarios: (1) all backcross individuals are fitted by a given QTL genotype-specific TPC with a large coefficient of determination (*R* ^2^ = 0.9–1.0), (2) a half of individuals are fitted by a large coefficient of determination (*R* ^2^ = 0.9–1.0), whereas the other half fitted by a low coefficient of determination (*R* ^2^ = 0.5–0.6), and (3) all individuals are fitted by a low coefficient of determination (*R* ^2^ = 0.5–0.6). Table 2 tabulates the means and SEs of the MLEs for the QTL position, curve parameters and covariance-structuring parameters. As expected, the accuracy and precision of parameter estimates increases with a higher proportion of individuals that can be better fitted by TPCs (see also Fig. 5). But even if all individuals have a modest coefficient of determination, the model can still provides reasonable parameter estimation.

**Table 2:** The averaged MLEs of the QTL position, curve and AR(1) parameters and their standard errors (given in parentheses) for different simulation scenarios for a hotter-colder QTL under *n* = 200 and *H* ^2^ = 0.4 based on 100 simulation replicates. The true parameters are *a*_1_ = *a*_0_ = 1, *b*_1_ = *b*_0_ = 0.2, *d*_1_ = *d*_0_ = 10, *c*_1_ = 5.0, *c*_0_ = 3.5, σ^2^ = 0.036, and ρ = 0.6.

Scenario	QTL Location	Genotype *Qq*				Genotype *qq*				AR(1) parameter	
		*â*_1_	*bˆ*_1_	*ĉ*_1_	*dˆ*_1_	*â*_0_	*bˆ*_0_	*ĉ*_0_	*dˆ*_0_	σˆ^2^	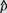
1	31.52 (1.53)	1.17 (0.03)	0.17 (0.02)	5.02 (0.04)	9.88 (0.03)	1.16 (0.03)	0.17 (0.02)	3.46 (0.04)	9.89 (0.03)	0.036 (0.002)	0.569 (0.021)
2	32.24 (3.00)	1.21 (0.05)	0.19 (0.03)	5.07 (0.08)	9.89 (0.07)	1.22 (0.05)	0.18 (0.03)	3.41 (0.08)	9.88 (0.05)	0.158 (0.005)	0.097 (0.025)
3	31.46 (4.15)	1.23 (0.05)	0.21 (0.04)	5.07 (0.10)	9.90 (0.06)	1.25 (0.05)	0.20 (0.03)	3.40 (0.09)	9.89 (0.05)	0.281 (0.007)	0.046 (0.024)

Note: Scenario 1–the coefficients of determination (*R* ^2^) equal to 0.9–1.0 for all individuals; Scenario 2–*R* ^2^ = 0.9–1.0 for a half of individuals and *R* ^2^ = 0.5–0.6 for the other half; Scenario 3–*R* ^2^ = 0.5–0.6 for all individuals.

## Discussion

Growth is an integrative process that involves digestion, absorption, assimilation, metabolic expenditure and excretion [28], [29]. All of these functions are mediated by enzymatic activities that are largely affected by temperature [30], [31]. Ultimately, thermal regimen emerges as the main factor controlling the growth rate of an organism [7], [8]. An accurate description of thermal dependence of any aspect of organismal performance should include three critical parameters: (1) temperature or range of temperatures for maximal performance (i.e. optimum), (2) thermal performance breadth (range of temperatures in which performance is above certain level) and (3) tolerance zone or range of above-zero performance [7], [32], [33]. Extensive studies have been carried out to establish an empirical model for specifying the relationship between growth rate and temperature in a variety of organisms [8], [10] and integrate it into the evolutionary and developmental context of adaptation [7], [8], [10], [33]. However, further incorporation of thermal sensitivity into evolutionary studies is limited by our poor understanding of the genetic machinery of this phenomenon. To our knowledge, no analytical model has been available to detect and characterize specific quantitative trait loci (QTL) that control thermal performance curves (TPC) based on their underlying mathematical functions.

Thanks to functional mapping, a general framework constructed to map QTL for quantitative traits that undergo developmental changes [19], [20], we are now able to derive an analytical model for mapping TPCs by implementing the biological principle of the thermal sensitivity. The new model includes two components. First, it integrates mathematical equations that specify the shape and process of TPCs into a statistical framework for QTL mapping, thus increasing the biological relevance and statistical power of the model. Second, because of the autocorrelation between longitudinal measures [22], parametric modeling of the structure and pattern for the covariance matrix increases the robustness of the model. Although a similar analytical principle of functional mapping has been used for its derivation process, the new model is different from the original model in the aspects as follows.

First, the new model embeds fundamental ideas of thermal sensitivity within QTL mapping, allowing for the characterization of different types of QTL that contribute to different modes of variation in TPC. Variation in TPC may be due to three different modes, hotter-colder, faster-slower and generalist-specialist [8]. Empirical studies suggest that these modes play different roles in affecting TPC differentiation in a population [10]. These roles can now be discerned by our model through the detection of the underlying genetic control mechanisms due to specific QTL. In this article, we propose a quantitative procedure for testing the existence and effect of so-called hotter-colder, faster-slower and generalist-specialist QTL on thermal performance. Second, the new model has for the first time provided a general framework in which development and genetics can be integrated with ecology to further and deepen the idea of Eco-Devo, aimed to study the developmental mechanism of ecological processes [34], [35]. In a couple with real genetic and phenotypic data to be collected in the design of this study, this model can be expected to push ecological genetic studies into a level at which a detailed picture of the developmental machinery of adaptation and evolution can be clearly elucidated. The utilization of the new model is validated through extensive simulation studies under different conditions that are faced in practice.

In this article, we limited our analysis to the mean rates of growth during a time course at individual temperatures. This treatment has simplified our modeling and calculation, but has ignored the role of development in the regulation of TPC differentiation. The biological relevance of our model can be enhanced by incorporating the growth equation into the mean vector. As a universal phenomenon, growth follows a rule that can be described by mathematical functions derived on the basis of the goodness-of-fit of observational data [36] or from fundamental biological principles [37]. If a logistic equation is used to describe growth trajectory, we can estimate the growth curve parameters for each QTL genotype and test how the detected QTL for TPC exerts its pleiotropic effect on time-dependent growth. This integrative model is supposed to be in a better position to unravel the genetic and developmental mechanisms of ecological adaptation within the *Evo-Devo* and *Eco-Devo* contexts [38].

In statistics, this model can be modified or extended to be more powerful. For the sake of description, the model was proposed on the basis of simple interval mapping [13]. But it is straightforward to incorporate composite interval mapping [14], [15] into the model, increasing the mapping resolution of linked QTL on the same chromosome. Composite interval mapping combines the idea of interval mapping and partial regression analysis with markers outside the test interval, minimizing the impacts of all those QTL residing outside the interval. As pointed out by Yang et al. [39], however, parametric fitting of individual marker effects will inhibit the implementation of composite interval mapping for dynamic traits. While a parametric method is used for interval mapping, partial regression analysis with other markers as co-factors can be effectively constructed by a nonparametric approach. The deployment of composite interval mapping will allow our model to precisely characterize the QTL that regulate thermal performance trajectories in additive or interactive manners. The computer code to perform linkage disequilibrium analyses can be requested from the corresponding author (rwu@stat.ufl.edu).

## Appendix

In what follows, we derive the log-likelihood functions used to estimate the parameters 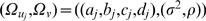. The symbol ' denotes the estimates of parameters from the previous step.
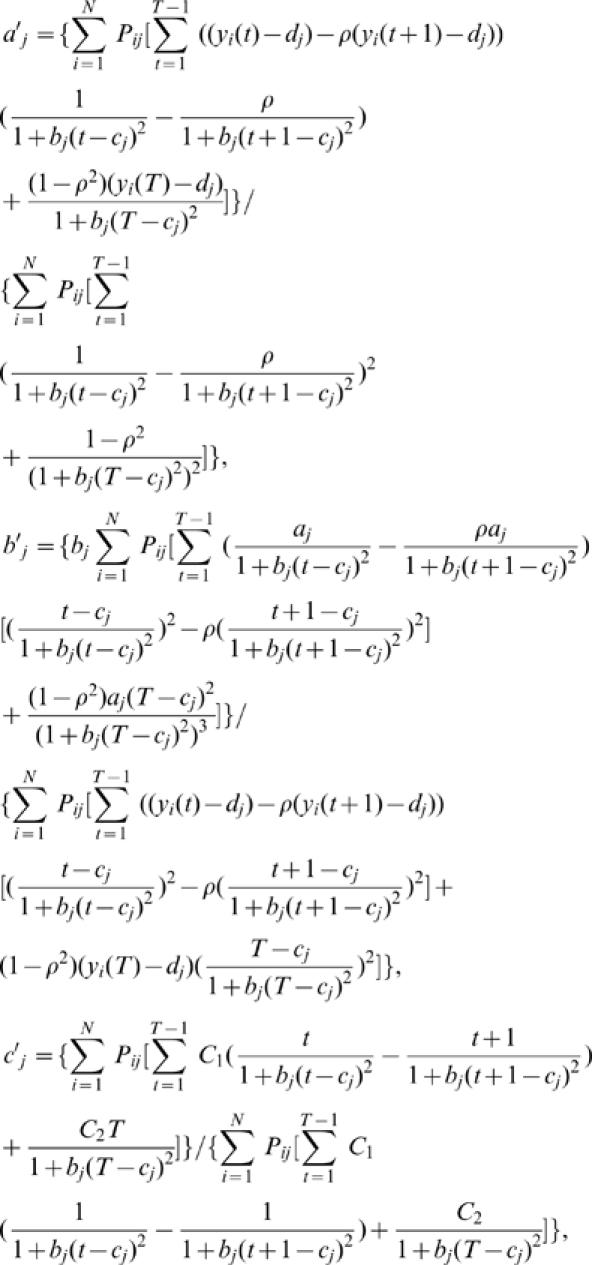
where
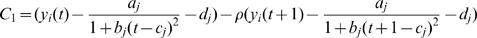
and
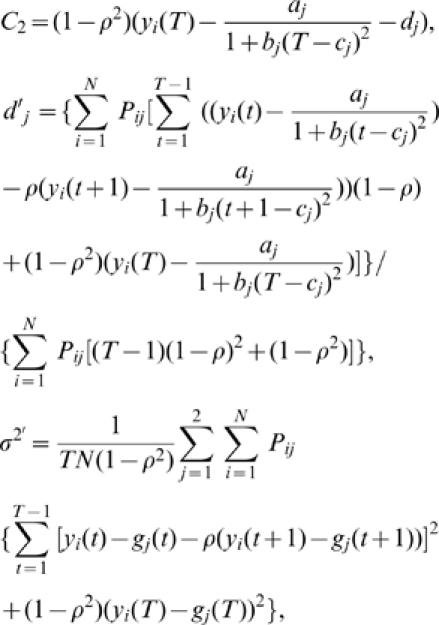
and
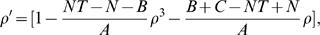
where